# Enhancing long-range depth estimation via heterogeneous CNN-transformer encoding and cross-dimensional semantic fusion

**DOI:** 10.1038/s41598-026-36755-0

**Published:** 2026-02-17

**Authors:** Yunhao Chen, Qian Yin, Li Zhao, Jianlong Wang, Sida Zhou, Jianing Tang

**Affiliations:** 1 School of Electrical and Information Engineering, Yunnan Minzu University, Kunming, 650500 China; 2 Yunnan Key Laboratory of Unmanned Autonomous Systems, Kunming, 650500 China

**Keywords:** Monocular depth estimation, Dual-branch encoder, Feature fusion, Attention mechanism, Long-range depth estimation, Engineering, Mathematics and computing

## Abstract

Monocular depth estimation enables 3D scene reconstruction from a single 2D image, offering a cost-effective solution widely applied in autonomous driving and UAVs. However, existing deep neural networks often fail to balance local texture details with global contextual information, leading to significant inaccuracies in distant-region depth prediction. To address this challenge, we introduce a novel monocular depth estimation framework featuring a heterogeneous encoder and a Cross-dimensional Semantic Fusion (CSF) module. The heterogeneous encoder integrates the initial convolutional layers of ResNet-50 with the hierarchical attention mechanism of Swin Transformer to efficiently capture both local details and long-range dependencies. Specifically targeting the characteristics of distant objects—low pixel occupancy but high semantic relevance—the CSF module enhances feature aggregation in the decoder through multi-scale interactions and spatial-channel coupling. Additionally, the decoder incorporates a Depth-Separable Upsampling Block (DSUB) and a Multi-scale Self-Attention (MSA) module to refine detail restoration and ensure spatial consistency. Experiments validate the superiority of our method. On the KITTI dataset, it achieves leading results: 0.050 Abs-Rel, 2.107 RMSE, and a long-range error of 0.2725. The SUN RGB-D dataset demonstrates strong generalization with an Abs-Rel of 0.142. This framework significantly advances long-range depth estimation research and shows broad application prospects.

**Supplementary Information:**

The online version contains supplementary material available at 10.1038/s41598-026-36755-0.

## Introduction

Monocular depth estimation aims to recover 3D scene structure from a single image, offering a cost-effective and flexible solution for applications such as robotic navigation and UAV control. Existing methods perform well in short- and mid-range scenarios where visual cues are abundant; however, their performance markedly degrades in long-range estimation tasks, where distant objects appear smaller, with sparser textures and blurrier contours. This poses potential risks to real-world applications such as UAV obstacle avoidance.

Monocular depth estimation has garnered extensive attention due to its low cost and high flexibility. By leveraging its powerful hierarchical feature extraction capabilities, Convolutional Neural Networks have been effectively applied in this field^1–6^, significantly improving the accuracy of depth estimation. Methods are generally categorized into supervised and self-supervised approaches: supervised methods use ground-truth depth data to achieve high accuracy and faster convergence, while self-supervised methods minimize photometric or geometric consistency losses during training. A key current challenge lies in efficient feature extraction, which is constrained by the limited receptive fields of CNNs. To address this, techniques such as the local planar guidance layer^7^ enhance local features, and bilateral depth encoders^8^ fuse multi-scale data. While these methods improve performance, they still exhibit shortcomings in depth continuity and edge sharpness, particularly in distant regions containing small objects and limited textures.

The emergence of Transformer^9,10^ has introduced new perspectives for monocular depth estimation by modeling global dependencies, excelling in long-range context, especially in distant regions. Recent works integrate Transformer modules: BinsFormer^11^ uses a Transformer decoder for adaptive depth bins and multi-scale interaction, FC-CRF^12^ enhances feature representation with a Transformer encoder. However, these models face challenges like computational inefficiency and weak spatial inductive bias for local details.

Balancing CNNs’ limited global modeling with Transformers’ weak local bias is a key challenge in monocular depth estimation. To address this issue, researchers have proposed several strategies. The first strategy focuses on enhancing global context modeling within convolutional architectures, which can be achieved in two ways: (1) improving the modeling capacity of CNNs through techniques such as large-kernel convolutions^13^, atrous convolutions^14^, and feature pyramids^15^ to extend receptive fields and enable better global feature aggregation. and (2) integrating attention modules into fully convolutional networks to facilitate global pixel-level interactions and enhance feature expressiveness^16^. The second strategy combines attention mechanisms with multi-scale feature fusion^4,17–19^. The third strategy involves hybrid CNN-Transformer architectures^20,21^, which aim to combine the local feature extraction capabilities of CNNs with the global dependency modeling strengths of Transformers. Although existing methods have achieved some progress, they still exhibit significant limitations in complex scenarios: the Depth Attention Volume Network^22^ lacks robustness in complex environments; the representation learning based on fixed pre-trained segmentation networks^23^ suffers from limited generalization capability; and hybrid architectures often incur prohibitively high computational costs in long-range tasks.

Overall, achieving accurate, robust, and efficient monocular depth estimation in complex environments—with a focus on long-range scenarios—remains an unresolved major challenge in the field. This performance bottleneck can be attributed to three interrelated fundamental factors: first, representational limitations in the feature extraction stage—the fixed-size patch embedding mechanism in visual Transformers^9,10^ tends to mix small-scale distant targets with background information, weakening their independent representations; while CNNs excel at capturing local texture details, they are limited by their finite receptive fields and cannot effectively model the global spatial relationships required for distant targets^21^. Second, inefficient interactions in the feature fusion stage—common strategies such as concatenation or element-wise addition^19,24^ struggle to reconcile the inherent conflicts between low-semantic high-resolution CNN features and high-semantic low-resolution Transformer features, resulting in edge blurring and depth discontinuities at the boundaries between near and long-range scenes. Third, scale-insensitivity in the optimization process—standard loss functions that treat all pixels equally allow prediction errors in distant, texture-sparse regions to disproportionately influence metrics like RMSE, thereby undermining the overall scene’s depth consistency and structural fidelity.

In response to the issues identified above, this work proposes a monocular depth estimation framework that integrates a heterogeneous encoder with cross-dimensional semantic fusion to address the challenge of inaccurate depth prediction in distant regions. The framework achieves state-of-the-art performance on benchmark datasets such as KITTI and NYU, while also demonstrating strong cross-dataset generalization capability. It maintains consistent performance across all distance ranges and achieves a favorable balance between estimation accuracy and inference speed. These advancements are attributed to the following three synergistic and innovative design components:

1. Heterogeneous hybrid encoder: A novel heterogeneous encoder is proposed, which integrates the initial convolutions of ResNet-50 with the hierarchical attention mechanism of Swin-Transformer to effectively and jointly capture local details and long-range dependencies.
2. Cross-dimensional Semantic Fusion (CSF) module: Targeting the characteristic of distant objects—characterized by low pixel proportion yet high semantic dependency—we further design a CSF module. It enhances the decoder’s feature aggregation capability through multi-scale interaction and spatial-channel coupling.
3. Attention-driven decoder: To address the issue of fine detail loss in depth maps caused by traditional upsampling methods, we propose an attention-driven decoding architecture. This architecture comprises a DSUB and an MSA mechanism, which effectively optimizes detail restoration and spatial consistency.

##  Related work

### Hybrid encoder architecture

CNNs excel at local feature extraction but are limited by narrow receptive fields for global reasoning. Transformers model long-range dependencies but lack local spatial bias. Hybrid architectures address this: TransDepth^21^ embeds Transformer modules into a ResNet framework, which is essentially a single-branch design, and employs a gated attention decoder for multi-scale feature integration, yet it struggles to preserve fine-grained details and maintain depth continuity. DPT-Hybrid^10^ and ASTransformer also adopt hybrid architectures, but they primarily rely on late-stage feature fusion, which may not fully exploit the complementary strengths of both architectures in capturing multi-scale contextual dependencies. Liu et al.^20^ propose a CNN-Transformer hybrid model that significantly improves prediction accuracy but introduces complexity and higher resource demands, with reduced performance in dynamic environments. Based on these insights, a novel heterogeneous hybrid encoder is introduced in this work to address these challenges and enhance the precision of depth estimation for distant objects.

In contrast, our proposed heterogeneous hybrid encode adopts a dual-branch encoder pathway structure: the CNN branch captures fine-grained spatial details, while the Transformer branch hierarchically models long-range dependencies. This design ensures that both local and global features are preserved and interact throughout the entire encoding process, rather than being merged only at later stages. Furthermore, unlike methods that simply stack Transformers, our encoder explicitly preserves low-level visual cues, which are crucial for estimating depth in distant, texture-sparse regions.

### Multi-scale feature fusion

To bridge the gap between CNN-based local detail extraction and Transformer-based global dependency modeling, existing research often employs multi-scale fusion methods to enhance feature interaction. However, most existing approaches rely on simple concatenation or additive fusion strategies, which struggle to effectively capture the complex interplay between local details and global context. Moreover, these methods typically operate in a single dimension, resulting in insufficient feature aggregation. This issue is particularly pronounced in long-range scenarios that simultaneously rely on fine-grained structures and holistic scene layouts.

For instance, Fu et al.^1^ introduced a discretization strategy and a multi-scale network, framing monocular depth estimation as an ordinal regression problem based on multi-level features, yet their method tends to lose precision in detailed areas. Lee et al.^7^ proposed the Local Planar Guidance layer, which enhances local depth features in the decoder but struggles to maintain global consistency. LapDepth^25^ utilizes a multi-scale encoder and a Laplacian pyramid with weight normalization to preserve local details, though its weak global modeling compromises overall accuracy. To address these limitations, we designed the CSF module. Unlike previous methods that perform only single-dimensional fusion, CSF supports multi-dimensional collaborative interaction, enabling the network to adaptively enhance relevant features based on local geometry and global semantics.

### Attention mechanisms

Attention mechanisms have become integral to monocular depth estimation due to their ability to dynamically adjust feature importance, improving both prediction accuracy and robustness to input variability. Techniques such as channel attention and multi-scale feature fusion have been widely applied. EncNet^26^ leverages channel attention to model global context, while CCNet^27^ adopts criss-cross attention to improve efficiency. Dual attention mechanisms^28^ and structured attention^12,29^ further enrich model representations through spatial and channel dependency modeling. PixelFormer^30^ incorporates a skip attention module to enhance critical region focus, improving accuracy at the expense of complexity. HQDec^31^ introduces adaptive axial attention to fuse fine-grained information but with increased computational cost. TransDepth^21^ proposes a gated attention decoder for improved fusion, yet faces challenges in computational efficiency and detail recovery. To address these issues, this work introduces a Multi-scale Self-Attention module, which adaptively refines decoder features across scales, significantly improving depth estimation accuracy while maintaining computational efficiency.

## Methods

**Fig. 1:**
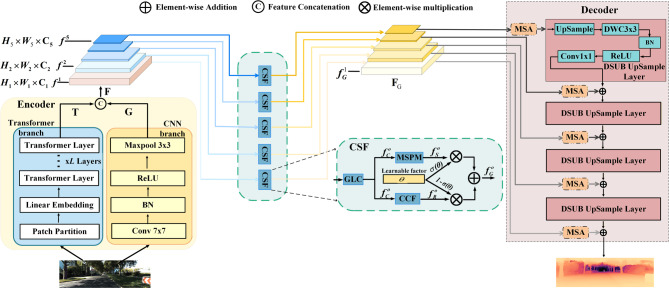
The network architecture includes three key components: an encoder with Transformer and CNN branches. The CSF module enhances interaction between Transformer features (T) and convolutional features (G) to improve feature aggregation, while the decoder, aided by DSUB (using nearest-neighbor upsampling).

The overall architecture of the proposed framework is illustrated in Fig. 1. It is designed to address the limitations of existing monocular depth estimation methods in long-range scenarios by combining heterogeneous encoding with cross-dimensional feature fusion. The framework consists of three core components. First, it adopts a dual-branch encoder, where the CNN branch extracts fine-grained local structures, and the Transformer branch models long-range spatial dependencies. Second, to effectively integrate the complementary strengths of both representations, a Cross-dimensional Semantic Fusion (CSF) module enables structured interaction across spatial and channel dimensions. Finally, an attention-driven multi-scale decoder progressively reconstructs high-resolution depth maps with improved global consistency and boundary precision.

### Heterogeneous hybrid encoder

Long-range depth estimation faces a core imbalance contradiction between local texture details and global spatial correlations, yet accurate depth estimation precisely requires the simultaneous consideration of these two types of key information. To address this, the heterogeneous encoder we designed achieves this goal by integrating the complementary strengths of CNNs and Transformers (see Fig. 1). Specifically, the CNN branch adopts the first convolutional block of ResNet-50 (denoted as R50-C1) to extract low-level spatial features, yielding a feature map$\mathrm{G} \in {{\mathbb{R}}^{{C_g} \times {H_g} \times {W_g}}}$that retains fine-grained structural details. The Transformer branch, built on Swin Transformer^9^, models cross-layer long-range dependencies and outputs four representative feature maps$\mathrm{T} \in \{ {t^n}\} _{{n=1}}^{N}$(where *N* denotes feature level). These CNN-extracted and Transformer-modeled features are further fused to generate a final five-layer multi-scale feature representation $\mathrm{F}=\{ {f^o}\} _{{o=1}}^{L},$ (where *L* denotes the number of multi-scale feature layers, and ${f^o} \in {{\mathbb{R}}^{{C_o} \times {H_o} \times {W_o}}}$for each layer).

### Cross-dimensional Semantic Fusion (CSF) module

After dual-branch encoding, to effectively integrate the long-range semantic information from the Transformer with the detailed structural features captured by the CNN, and to alleviate the decoder’s insufficient feature aggregation capability, we propose the Cross-dimensional Semantic Fusion (CSF) module. This module performs structured multi-level feature integration through three coordinated stages: Global-Local Channel (GLC) fusion, Multi-scale Spatial Prior Modeling (MSPM), and Cross-scale Context Fusion (CCF), as shown in Fig. 1. This design enables precise alignment between global context and local details, mitigating the common issue in traditional fusion schemes where distant semantic cues are suppressed by nearby dominant textures.

**Fig. 2:**
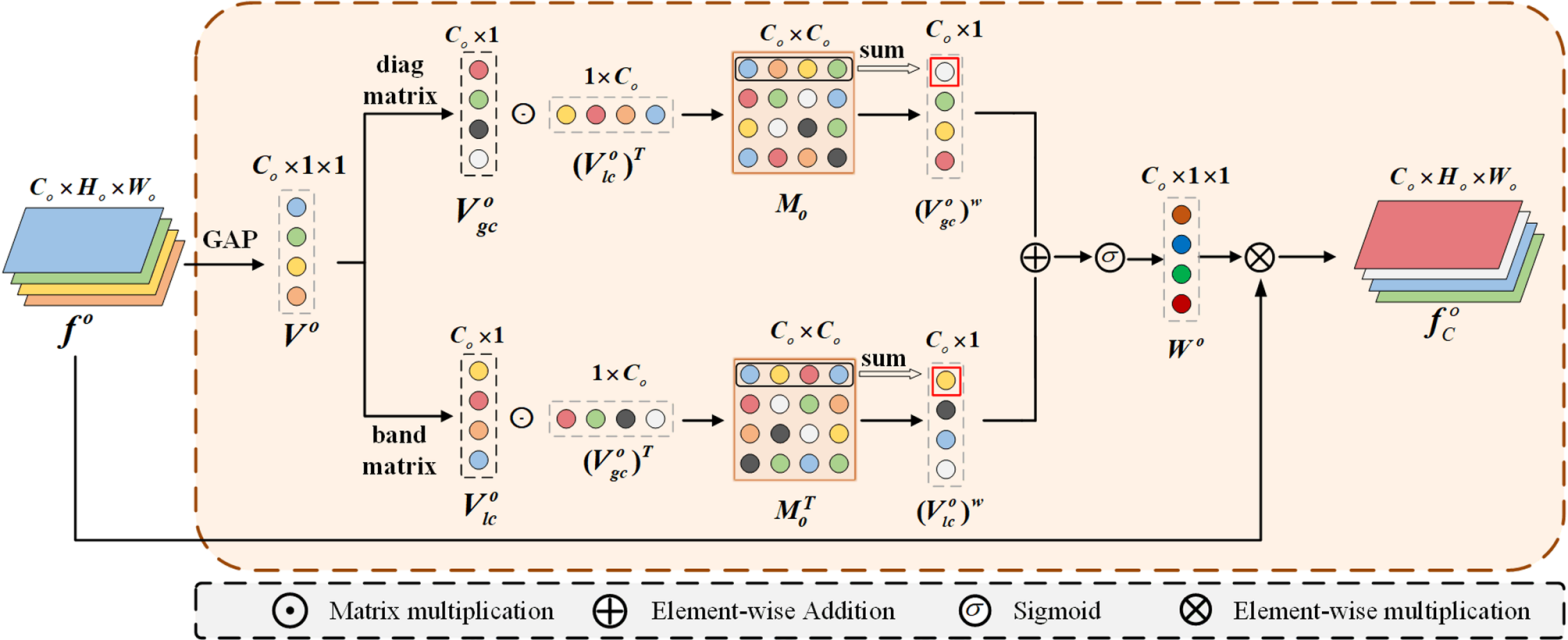
The GLC module. ${f^o}$represents the *o*-th layer among the *L-*layer multi-scale features output by the encoder, where $o \in [1,L]$.

For the feature ${f^o} \in {{\mathbb{R}}^{{C_o} \times {H_o} \times {W_o}}},o \in [1,L]$, the CSF module enhances its channel representation capability through the global-local channel fusion mechanism at the GLC stage (Fig. 2). A channel identifier ${V^o}$ is first formed via global average pooling (GAP). Then, a band matrix $B=[{b_1},{b_2},...,{b_k}]$and a diagonal matrix $D=[{d_1},{d_2},...,{d_{{C_{\mathrm{o}}}}}]$are employed to model local and global channel dependencies, respectively, thereby generating the channel weight vector. Specifically, the band matrix *B* is implemented via 1D convolution, where its kernel size *k* dictates the number of local neighboring channels involved in the interaction, thereby computing the local channel dependency $V_{{lc}}^{o}=\sum\limits_{{i=1}}^{{{k{\text{}}}}} {{V^o}\cdot {b_i}}$. The diagonal matrix *D* is implemented via 1 × 1 convolution to capture global information across all channels, and the corresponding global channel dependency $V_{{gc}}^{o}=\sum\limits_{{i=1}}^{C_{o}} {{V^o}\cdot {d_i}}$ is computed accordingly.

Subsequently, the outer product of these descriptors is then computed to form a correlation matrix ${M_o}=V_{{gc}}^{o}\cdot {(V_{{lc}}^{o})^T}$ and its transpose $M_{o}^{T}=V_{{lc}}^{o}\cdot {(V_{{gc}}^{o})^T}$. By performing element-wise summation on these two matrices, the global and local channel weights denoted as ${(V_{{gc}}^{o})^w}=\sum\limits_{j}^{{{C_o}}} {(M_{{o}}^{})_i,_j}$ and ${(V_{{lc}}^{o})^w}=\sum\limits_{j}^{{{C_o}}} {((M_{{{o}}}^{T}} )_i,_j),i \in 1,2,...,{C_o}$ are derived. These weights are then combined and passed through a Sigmoid activation function to generate the weighting coefficient ${W^o}={\mathrm{sigmoid}}({(V_{{lc}}^{o})^w}+{(V_{{{\mathrm{g}}c}}^{o})^w})$, which is used to complement the original feature ${f^o}$, yielding the channel-enhanced feature $f_{C}^{o}={W^o} \times {f^o}$. resulting in multi-scale features ${\mathrm{F}_\mathrm{C}}=\{ f_{C}^{o}\} _{{o=1}}^{L}$ (Fig. 1). This stage enables the network to simultaneously focus on local detail channels and global semantic channels, providing rich contextual information for subsequent spatial modeling.

**Fig. 3:**
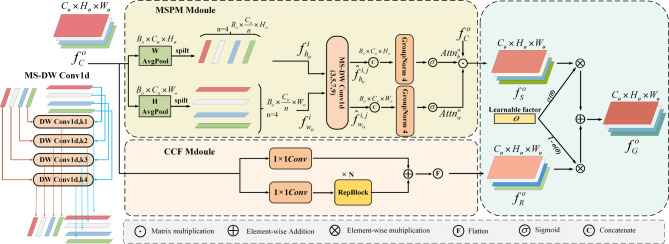
The MSPM and CCF modules, MS-DW Conv1d stands for multi-receptive-field shared depthwise 1D convolutions with kernel sizes of 3, 5, 7, and 9.

Considering that in monocular depth estimation, scene depth distribution often exhibits distinct directional structural priors—for example, roads and buildings primarily extend horizontally, while objects such as trees and pedestrians display clear vertical contours—in Stage 2, we propose the MSPM (see Fig. 3). This model performs directional decomposition and multi-receptive-field fusion on the GLC output feature $f_{C}^{o} \in {{\mathbb{R}}^{{C_o} \times {H_o} \times {W_o}}}$ in the spatial dimension. Specifically, segment-wise average pooling is applied along the height and width directions, resulting in two one-dimensional sequences $f_{h}^{o} \in {B_o} \times ({C_o}/n) \times {H_o}$ and $f_{w}^{o} \in {B_o} \times ({C_o}/n) \times {W_o}$, each divided into *n* segments. For the *i*-th sub-feature, MS‑DW Conv1d (Multi‑Receptive‑Field Shared Depthwise 1D Convolutions) with kernel sizes of 3, 5, 7, and 9 is employed to extract feature representations in the height and width directions, respectively. By combining the features from the horizontal and vertical directions and normalizing them, the spatial attention weights are obtained:

1 $$\begin{gathered} A{\mathrm{ttn}}_{h}^{o}=\sigma (GN(Concat(\hat {f}_{{{h_o}}}^{i}) \in {{\mathbb{R}}^{{B_o} \times {C_o} \times {H_o}}})) \in {{\mathbb{R}}^{{C_o} \times {H_o} \times {W_o}}} \hfill \\ A{\mathrm{ttn}}_{w}^{o}=\sigma (GN(Concat(\hat {f}_{{{w_o}}}^{i}) \in {{\mathbb{R}}^{{B_o} \times {C_o} \times {W_o}}})) \in {{\mathbb{R}}^{{C_o} \times {H_o} \times {W_o}}} \hfill \\ \end{gathered}$$

These weights modulate the input feature $f_{C}^{o}$to generate a spatially refined representation, and we denote this set of features as${\mathrm{F}_\mathrm{S}}=\{ f_{S}^{o}\} _{{o=1}}^{L}$. This stage explicitly models the geometric structures within the scene, enhancing the perception capability for long-range targets such as roads and buildings. The spatially refined representation is expressed as:

2 $$f_{S}^{o}=Attn_{h}^{o} \times Attn_{w}^{o} \times f_{C}^{o}$$

To integrate shallow details and deep semantic information, we designed the CCF structure to process the output of GLC in parallel. One branch employs a 1 × 1 convolution to retain shallow features, while the other utilizes stacked *RepConv* blocks^32^ to capture deep channel interaction features, which are then fused through the following operation:

3 $$\begin{gathered} f_{R}^{o}=Con{v_1}(f_{C}^{o})+RepBloc{k_N}(RepBloc{k_{N-1}} \hfill \\ (...RepBloc{k_2}(RepBloc{k_1}(Con{v_1}(f_{C}^{o})))...)) \hfill \\ \end{gathered}$$

The multi-scale feature from CCF is denoted as ${\mathrm{F}_\mathrm{R}}=\{ f_{R}^{o}\} _{{o=1}}^{L}$. To adaptively balance the contributions of the spatially enhanced feature$f_{S}^{o}$and the cross-scale enriched feature$f_{R}^{o}$, in the third stage, we introduce a learnable scalar parameter σ(*θ*), which is passed through a Sigmoid activation to generate a soft weighting coefficient. The final output feature is computed as a convex combination of the two branches:

4 $$f_{G}^{o}=\sigma (\theta ) \times f_{S}^{o}+(1 - \sigma (\theta )) \times f_{R}^{o}$$

The CSF module, through the coordinated optimization of channel, spatial, and scale dimensions, remarkably enhances the representational capacity of features in long-range, low-texture regions. The final output is denoted as ${\mathrm{F}_\mathrm{G}}=\{ f_{G}^{o}\} _{{o=1}}^{L}$, which effectively alleviates the issue where distant features are overshadowed by strong nearby textures, thus providing structurally clear and semantically rich feature representations for the decoding stage.

### Attention-driven decoder

Traditional upsampling often results in the loss of fine details in depth maps. Our attention-driven decoder (see Fig. 1, right) addresses this by efficiently recovering high-resolution depth maps while preserving structural integrity. It integrates two key innovations: (1) a DSUB: This lightweight module uses nearest-neighbor interpolation, followed by a 3 × 3 depthwise separable convolution, batch normalization, ReLU activation, and a 1 × 1 convolution. By replacing standard convolutions, it reduces computational overhead while retaining contextual details. (2) MSA Module (based on Ref.^29^: Long-range depth map upsampling has two key issues, namely regional misalignment and computational explosion caused by high-dimensional channels. To address these problems, we design the MSA module based on the “channel grouping + batch dimension reuse” paradigm. This paradigm balances the independence of each channel group and the sharing of convolution weights, which not only reduces the complexity of attention operations but also optimizes the decoder features.

MSA module operates on the five-feature map output from the CSF module and aims to improve the expressive capacity of the upsampled representations through attention-based refinement. Specifically, the features map$f_{G}^{o},o \in [1,L]$is divided into *S* channel groups. The feature of the *s*-th group is denoted as:

5 $${(f_{G}^{o})_s}=G{\mathrm{roupSplit(}}f_{G}^{o}{\mathrm{,}}S{{\mathrm{)}}_s},s=1,2...,S$$

**Fig. 4:**
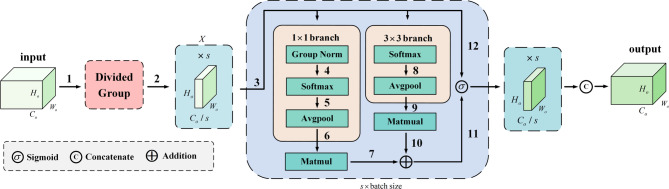
The MSA model. “$s \times \mathrm{b}\mathrm{a}\mathrm{t}\mathrm{c}\mathrm{h} \mathrm{s}\mathrm{i}\mathrm{z}\mathrm{e}$” means treating the group number *s* as the batch dimension, having *s* sub-features go through 1 × 1 branch and 3 × 3 branch, and Matmul processing in parallel, then finally concatenating and fusing these processed sub-features.

To allow the grouped feature maps to share a single set of convolutional weights, we cast the group count s into the batch dimension, resulting in *s* smaller feature maps denoted as *X*, each with dimensions $(s \times B) \times {C_o}/s \times {H_o} \times {W_o},o \in [1,L]$. Each group is then processed through a dual-branch convolutional design using 1 × 1 and 3 × 3 kernels to extract complementary multi-scale information. The outputs of both branches are fused via matrix multiplication to generate attention weights, which capture both global contextual relevance and pixel-level dependencies. Through this process, the MSA module significantly improves the spatial and contextual richness of upsampled features, offering better support for high-quality depth reconstruction. The dimensional configurations of each sub-module depicted in Fig. 4 are provided in Table 1.

The decoder is supervised using the following scale-aware logarithmic depth loss:

6 $${L_{pixel}}=\alpha \sqrt {( 1/T) \cdot \sum\limits_{i} {( \mathrm{l}\mathrm{o}\mathrm{g}{{\tilde {\mathrm{d}}}_\mathrm{i}}-\mathrm{l}\mathrm{o}\mathrm{g}{\mathrm{d}_\mathrm{i}}{) ^2}} - ( \lambda /{T^2}) \cdot {{(\sum\limits_{i} ( \mathrm{l}\mathrm{o}\mathrm{g}{{\tilde {\mathrm{d}}}_\mathrm{i}}-\mathrm{l}\mathrm{o}\mathrm{g}{\mathrm{d}_\mathrm{i}}{) ^2})}^2}}$$

where ${d_i}$and ${\tilde {d}_i}$denote the ground truth and predicted depths, respectively, and *T* is the number of valid pixels. Empirically, we set $\lambda =0.85$ and$\alpha =10$for all experiments.

**Table 1:** The output size of each module in the MSA.

Block Id	Output Size	Block Id	Output size
1	*C*_*o*_ *× H*_*o*_ *× W*_*o*_	7	1*×H*_*o*_*×W*_*o*_
2	(*C*_*o*_*/s*) *× H*_*o*_ *× W*_*o*_	8	(*C*_*o*_*/s*) *× H*_*o*_ *× W*_*o*_
3	(*C*_*o*_*/s*) *× H*_*o*_ *× W*_*o*_	9	(*C*_*o*_*/s*) *×*1* × *1
4	(*C*_*o*_*/s*) *× H*_*o*_ *× W*_*o*_	10	1 *× H*_*o*_ *× W*_*o*_
5	(*C*_*o*_*/s*) *× H*_*o*_ *× W*_*o*_	11	1 *× H*_*o*_ *× W*_*o*_
6	(*C*_*o*_*/s*) *×* 1 *×* 1	12	1 *× H*_*o*_ *× W*_*o*_

## Experiment

### Datasets and evaluation metrics

In this study, we use three widely adopted depth estimation datasets: KITTI^33^, NYU-Depth-v2^34^, and SUN RGB-D^35^, covering diverse scenarios. KITTI (93 K + RGB/depth pairs) is a standard outdoor benchmark, while NYU-Depth-v2 (640 × 480 RGB-D) represents indoor scenes. SUN RGB-D (10,335 multi-sensor indoor scenes) is designed for indoor RGB-D tasks.

Evaluation Metrics: To evaluate the performance of depth estimation models, we adopt several standard metrics widely used in depth estimation: Absolute Relative Error (Abs-Rel), denoted as REL; Root Mean Square Error (RMSE); log10 and RMSE log; Accuracy under threshold metrics: δ < 1.25, δ < 1.25^2^, δ < 1.25^3^, denoted as δ1, δ2, and δ3, respectively.

Boundary accuracy evaluation: To evaluate depth prediction accuracy along object boundaries, we adopt the boundary metric method from^3^, which applies the Sobel operator to both predicted and ground truth depth maps to extract boundary pixels—those with gradient magnitudes above a threshold$\delta \in \{ 0.25,0.5,1.0\}$.We compute true positive (TP), false positive (FP), and false negative (FN) boundary pixels to derive Precision, Recall, and F1-score, assessing the model’s ability to preserve structural accuracy in depth predictions.

Long-range region error assessment: For long-range region error assessment, we use the long-range region metric^16^ to measure depth prediction accuracy for distant objects. Both predicted (*P)* and ground truth (*G)* depth maps are divided into *m* × *m* blocks ($m \in \{ 8,16,32\}$), with each block’s average depth calculated. The block with the maximum average depth in the predicted map is denoted as${P_{\hbox{max} }}({u_1},{v_1})$, and in the ground truth map as${G_{\hbox{max} }}({u_2},{v_2})$, where$( {u_1},{v_1}) {\mathrm{and}} ( {u_2},{v_2}) \in {N^+}$. The spatial localization error between the predicted and ground truth long-range regions is then defined as

7 $$E=\frac{1}{{{N_{test}}}}\sum {_{{n=1}}^{{{N_{test}}}}} \frac{1}{{m\sqrt 2 }}||P_{{\hbox{max} }}^{n} - G_{{\hbox{max} }}^{n}|{|_2}$$

where${N_{test}}$is the count of test images, $1/(m\sqrt 2 )$is used to normalize the distance error, and *n* is the *n*-th depth map.

### Experimental setup and parameter settings

All training and evaluation were conducted on the Ubuntu 20.04 operating system equipped with NVIDIA RTX A6000T GPUs. During the training process, the batch size was set to 2, the initial learning rate was 1e-4, and the total number of iterations was 38,400, with model evaluation performed every 800 iterations. A linear learning rate warm-up strategy^8^ was adopted in the first 30% of training iterations, and a cosine annealing decay schedule^9,10^ was applied for the remaining iterations. The window size of Swin-Transformer was set to 16; specifically, the learning rate of the Transformer module was configured as 6e−5, while the learning rate of the CNN branch was set to 1e−4. The encoder was first pre-trained on the ImageNet dataset and then fine-tuned on depth estimation datasets. In a single-node environment with 3 RTX A6000T GPUs, the overall training took approximately 5 h; the model has 330 million parameters and 580 GFLOPs of computational complexity.

### Comparison of experimental results

Our approach achieves higher accuracy in estimating the depth of distant objects, while maintaining reliable performance at short and medium ranges. To demonstrate its effectiveness and generalization across both indoor and outdoor environments, we conduct comprehensive comparisons with several state-of-the-art monocular depth estimation models on the KITTI and NYU-Depth-v2 datasets. Furthermore, to evaluate cross-dataset generalization, we directly apply the model pre-trained on NYU-Depth-v2 to the SUN RGB-D dataset without any fine-tuning.

**Fig. 5:**
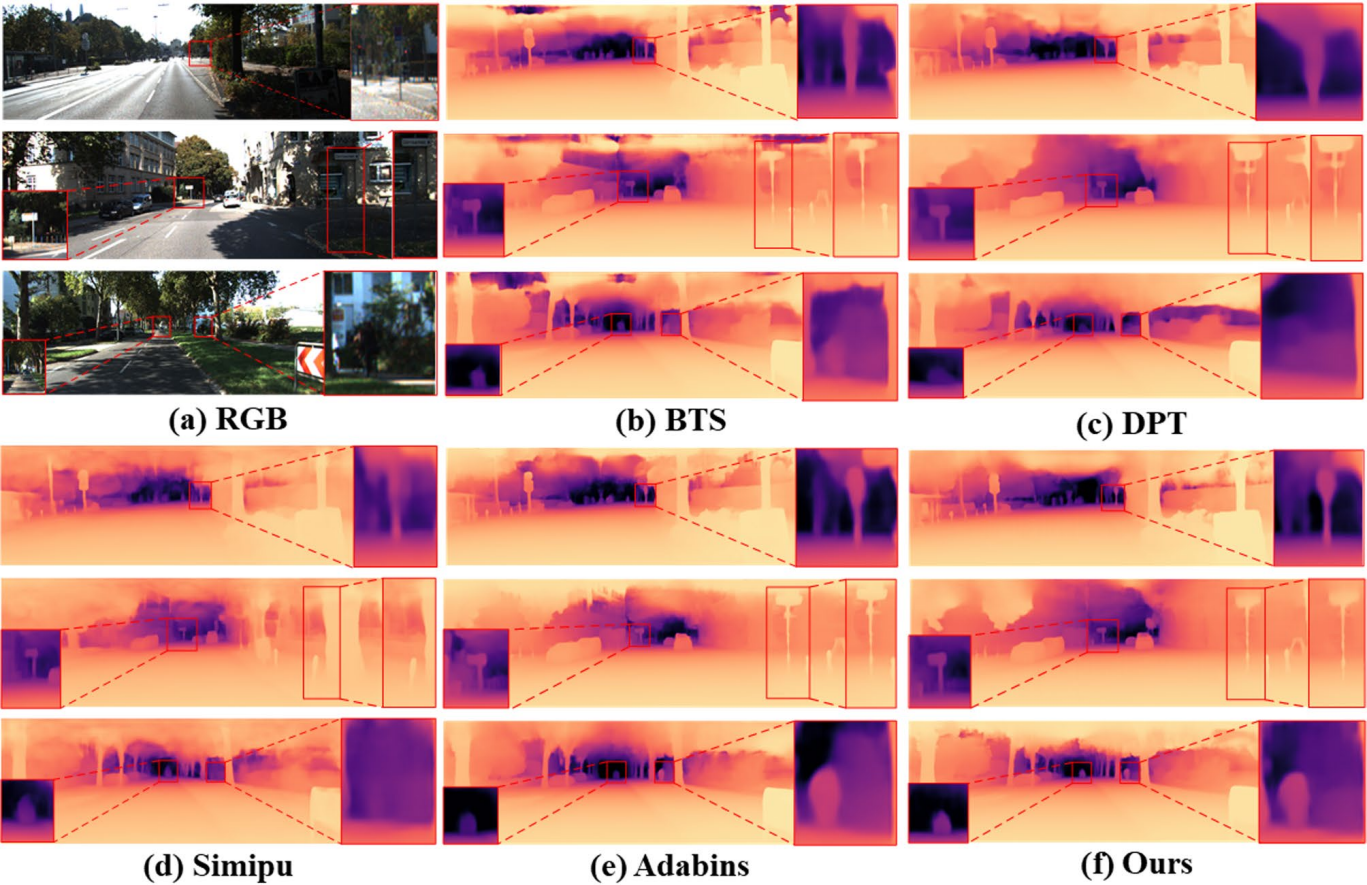
Qualitative comparison on the KITTI dataset shows our method outperforms others. In the first two input images, our approach accurately estimates distant signboard depths. In the third image, it precisely captures distant vehicles and cyclists with clear, coherent depth contours.

KITTI results: Our method was trained on 26 K images and evaluated on 697 test samples. As shown in Table 2, it achieves the best overall performance among all compared methods, with REL and RMSE reduced to 0.050 and 2.107, respectively. The slightly higher RMSE may be influenced by the metric’s sensitivity to large absolute deviations, rather than indicating an actual performance gap. In fact, our method consistently outperforms others across almost all standard metrics, affirming its strength in long-range depth estimation. Qualitative comparisons in Fig. 5 further highlight these advantages. Unlike the blurred edges and background blending seen in BTS and DPT, our method preserves sharp object boundaries and aligns depth values more accurately with real-world contours. Compared to Simipu, which struggles with adjacent distant vehicles, and AdaBins, where cyclists often appear visually detached from the scene, our model clearly resolves fine structural details—such as vehicle outlines and cyclist posture—demonstrating its strength in handling challenging long-range scenarios.

**Table 2:** Performance comparison on the KITTI dataset within 0–80 m depth range. Figures reported are from the corresponding original papers. The best results are shown in bold, and the second-best results are italics. ↑ means higher is better, ↓ means lower is better. C_*i*_ represents the *i*-th block of the ResNet-50 network.

Method	Backbone	δ1↑	δ2↑	δ3↑	REL↓	RMSE log↓	RMSE↓	Sq_Rel↓
CLIPMDE^2^	CLIP	0.550	0.830	0.938	0.303	0.119	6.322	–
Hu et al.^36^	CLIP-B	0.312	0.569	0.739	0.384	–	12.29	4.661
AMENet^4^	Hybird-ViT	0.851	0.977	0.988	0.112	0.115	4.561	1.121
DORN^1^	ResNet-101	0.932	0.984	0.995	0.072	0.120	2.727	0.307
Yin et al.^32^	ResNeXt-101	0.938	0.990	0.998	0.072	0.117	3.258	–
Monodepth2^37^	ResNet-50	0.883	0.962	0.982	0.110	0.831	4.642	0.831
BTS^7^	DenseNet-161	0.955	0.933	0.998	0.060	0.096	2.798	0.245
Simipu^38^	–	0.949	0.993	0.998	0.067	–	2.592	0.235
TransDepth^21^	ViT-B + ResNet-50	0.956	0.994	**0.999**	0.064	0.098	2.755	0.252
ASTransformer^39^	ViT-B	0.963	0.995	**0.999**	0.058	0.089	2.685	–
AdaBins^8^	EFN-B5 + ViTmini	0.964	0.995	**0.999**	0.058	0.089	2.360	0.190
DPT-Hybird^10^	ViT-B + R-50-C1C2	0.959	0.995	**0.999**	0.062	0.092	2.573	–
Yu et al.^40^	Swin-L	*0.972*	*0.996*	**0.999**	*0.054*	*0.081*	2.134	–
DDP-step3^41^	Swin-T	0.969	*0.996*	**0.999**	*0.054*	0.083	2.172	0.356
CaBins^42^	CLIP-B	0.964	0.995	**0.999**	0.057	0.088	2.322	0.186
CLIP2Depth^43^	CLIP-B	0.938	0.990	0.998	0.074	–	2.948	0.303
FutureDepth^44^	ResNet34	0.965	–	–	*0.054*	0.087	**2.016**	*0.179*
Xiang et al.^45^	ResNet-50	0.966	0.995	**0.999**	0.057	0.089	2.376	0.196
MambaDepth^46^	Mamba	0.940	0.989	0.996	0.072	0.114	3.490	0.410
LapUNet^5^	ResNet-101	0.964	0.994	**0.999**	0.055	0.085	2.247	0.200
Ours	Swin-L + R50-C1	**0.975**	**0.997**	**0.999**	**0.050**	**0.078**	*2.107*	**0.151**

**Fig. 6:**
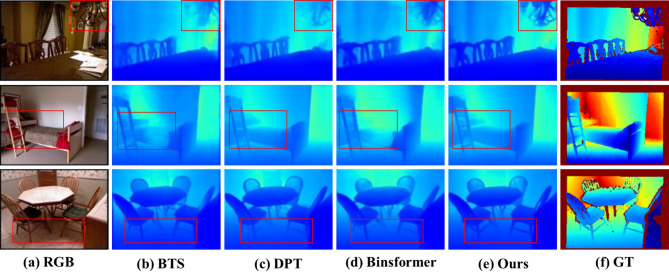
Qualitative comparison on the NYU Depth V2 dataset, demonstrating that our method accurately predicts fine edge contours of objects such as chandeliers and chairs, with clearer and more coherent results compared to other approaches.

NYU Results: On the NYU-Depth-v2 dataset, our model was trained on 50 K RGB-depth image pairs. The model first predicts depth maps at a resolution of 320 × 240, which are then upsampled to match the size of the ground truth annotations, and a standard center-cropping strategy is adopted for evaluation. As shown in Table 3, under the same Swin-Large backbone network, our method outperforms BinsFormer on most evaluation metrics (with only a slight underperformance on a few indicators), particularly excelling in mid- to short-distance regions. The key difference lies in the fact that BinsFormer relies entirely on the Transformer for global modeling, whereas our heterogeneous encoder retains the advantage of CNN in extracting local details at the lower layers. The qualitative comparison in Fig. 6 further demonstrates the superiority of our method in fine-grained boundary preservation and indoor structure reconstruction: compared to issues such as blurred lamp posts, fused chair contours, and missing bed frame structures in BinsFormer, our method accurately preserves fine edges, clearly delineates chair legs, and fully reconstructs bed frame structures, thereby ensuring the completeness of scene reconstruction.

**Table 3:** Performance comparison of different methods on the NYU-v2 dataset. The reported figures are from the corresponding original papers. The best results are shown in bold, and the second-best results are italics, ↑ means higher is better, ↓ means lower is better.

Method	Backbone	δ1 ↑	δ2 ↑	δ3 ↑	REL ↓	log_10_ ↓	RMSE ↓
DepthCLIP^47^	CLIP (zero-shot)	0.394	0.683	0.851	0.388	0.156	1.167
Hu et al.^36^	CLIP-B	0.428	0.732	0.898	0.347	0.140	1.049
CLIPMDE^2^	CLIP	0.465	0.776	0.922	0.319	0.139	0.970
DORN^1^	ResNet-101	0.828	0.965	0.992	0.115	0.051	0.509
Yin et al.^32^	ResNet-101	0.875	0.976	0.994	0.108	0.048	0.416
BTS^7^	DenseNet-161	0.885	0.978	0.994	0.110	0.047	0.392
ASN^24^	HRNet-48	0.890	0.982	0.996	0.101	0.044	0.377
TransDepth^21^	ViT-B + ResNet-50	0.900	0.983	0.996	0.106	0.045	0.365
ASTransformer^39^	ViT-B	0.902	0.985	0.997	0.103	0.044	0.374
AdaBins^8^	EFN-B5 + ViT-mini	0.903	0.984	0.997	0.103	0.044	0.364
DPT-Hybird^10^	ViT-B + R-50-C1C2	0.904	0.988	**0.998**	0.110	0.045	0.357
BinsFormer^11^	Swin-L	**0.925**	*0.989*	0.997	**0.094**	**0.040**	*0.330*
CaBins^42^	CLIP-B	0.886	0.978	0.996	0.120	0.050	0.401
RADepthNet^48^	ResNet-152	0.894	0.979	0.994	0.108	0.045	0.393
Xiang et al.^45^	ResNet-50	0.897	0.977	0.994	0.112	0.048	0.420
CLIP2Depth^43^	CLIP-B	0.900	0.983	0.996	0.100	0.042	0.379
Xia et al.^6^	ViT	0.895	0.988	0.996	0.109	-	0.351
Ours	Swin-L + R50-C1	*0.923*	**0.990**	**0.998**	*0.096*	**0.040**	**0.329**

**Fig. 7:**
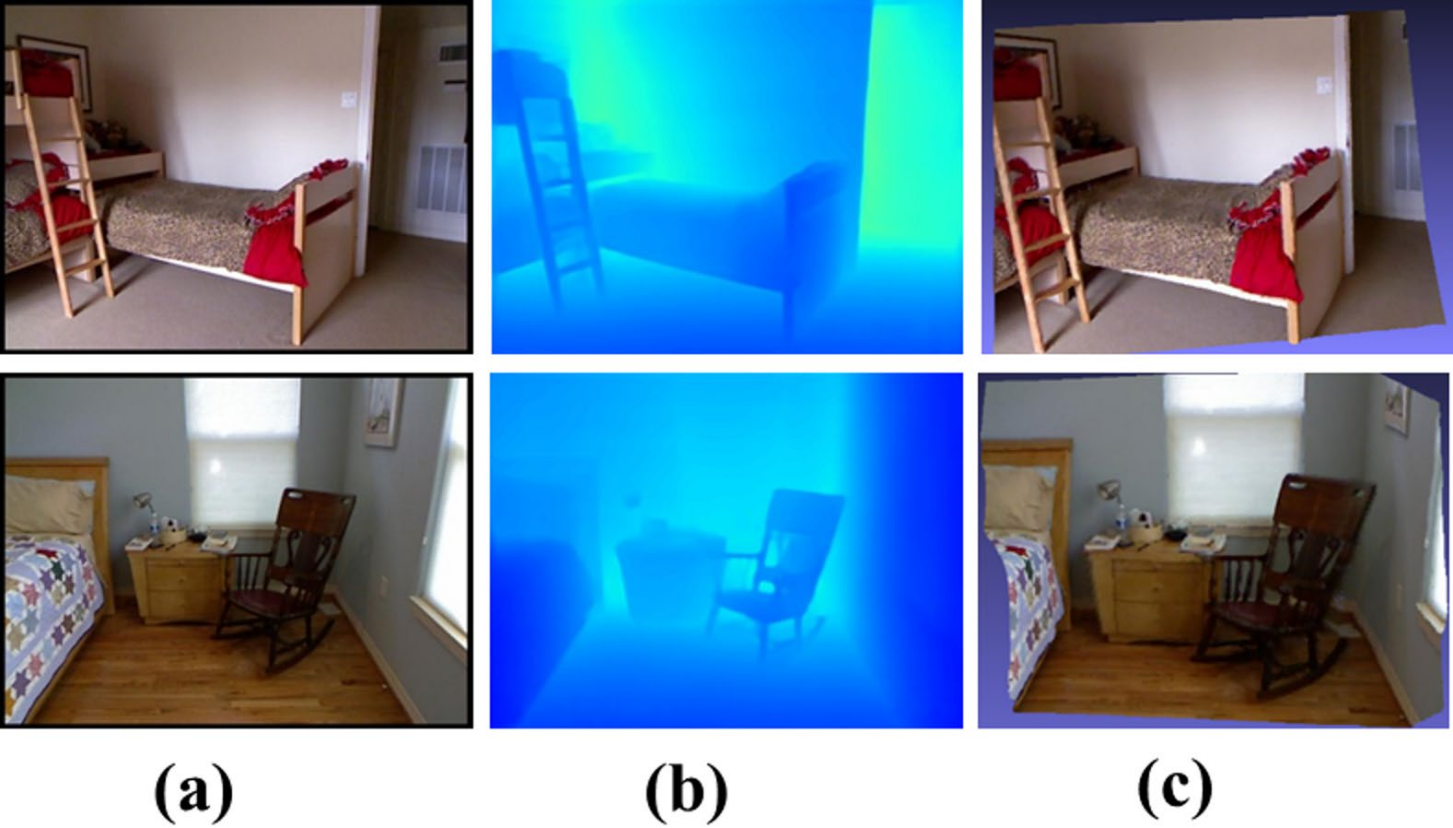
Visualization of 3D scene reconstruction results based on the NYU Depth V2 dataset. (**a**) Input RGB image. (**b**) Predicted depth map generated by our method. (**c**) 3D point cloud reconstructed from the predicted depth.

3D point cloud reconstruction: The pretrained depth estimation model predicts dense per-pixel depth maps from input RGB images. Using the camera intrinsics and a pinhole camera model, these depth maps are back-projected into 3D space to generate dense point clouds in the camera coordinate frame. RGB colors are then assigned to the corresponding 3D points, forming colored point clouds saved in PLY format for visualization (e.g., MeshLab). As shown in Fig. 7, the reconstructed scene—particularly the bed ladder and chairs—exhibits clear object contours and spatially consistent depth, demonstrating the effectiveness of our model in real-world 3D reconstruction applications.

**Fig. 8:**
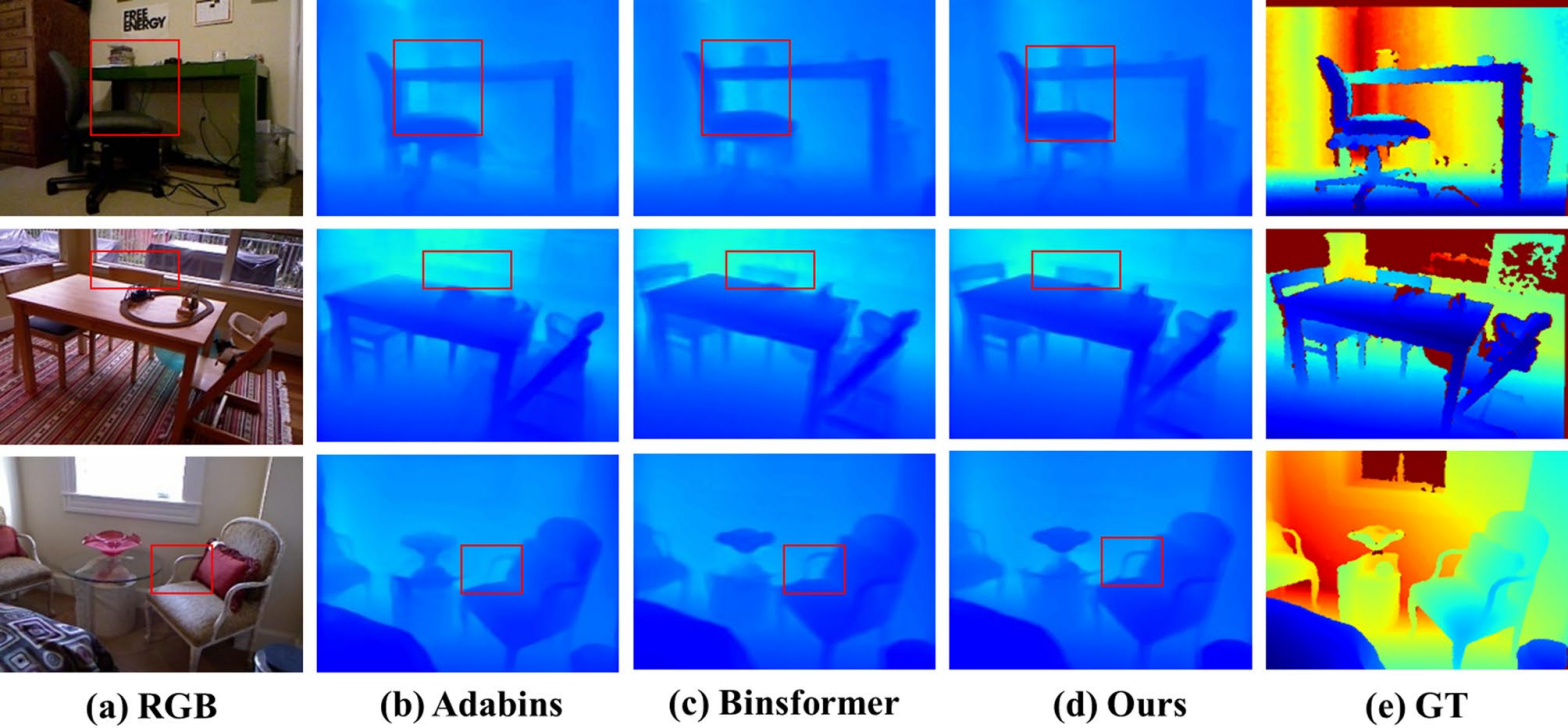
Qualitative comparison of different methods on the SUN RGB-D dataset, highlighting the effectiveness of our approach in accurately estimating object depths and fine details.

SUN-RGBD results: To assess the model’s generalization capability, we directly evaluated it on the SUN RGB-D dataset (comprising 5,050 indoor images with a maximum depth of 8 m), using the weights trained solely on NYU-Depth-v2 without any fine-tuning. As shown in Table 4, our method delivers competitive performance across all metrics and achieves the best overall balance. While BinsFormer achieves slightly better results on two individual indicators (δ1 and RMSE), our approach demonstrates more consistent accuracy across the full evaluation spectrum. The qualitative results in Fig. 8 further confirm the robustness of our method under diverse indoor conditions, with clear preservation of structural details and sharp object boundaries, even in scenes with complex geometry or challenging lighting.

**Table 4:** Cross-dataset evaluation of different depth Estimation methods trained on NYU and tested on SUN RGB-D (no fine-tuning).

Method	Backbone	δ1 ↑	δ2 ↑	δ3 ↑	REL ↓	RMSE ↓	log10 ↓
Chen et al.^49^	SEnet	0.757	0.943	0.984	0.166	0.494	0.071
BTS^7^	DenseNet161	0.740	0.933	0.980	0.172	0.515	0.075
Yin et al.^32^	ResNeXt-101	0.696	0.912	0.973	0.183	0.541	0.082
Adabins^8^	EFNB5 + ViT-mini	0.771	0.944	0.983	0.159	0.476	0.068
Binsformer^11^	Swin-L	**0.805**	*0.963*	*0.990*	*0.143*	**0.421**	**0.061**
Ours	Swin-L + R50-C1	*0.800*	**0.965**	**0.992**	**0.142**	*0.433*	**0.061**

Long-range region error comparison: To evaluate the accuracy of the model in depth estimation for long-distance regions, we conducted tests using the long-range region error metric on the KITTI test set. As shown in Table 5, our method outperforms other comparative models in this long-distance depth estimation task. Specifically, the implementation process is as follows: first, depth maps with a resolution of 1216 × 352 are divided into multiple *m*×*m* image patches ($m \in \{ 8,16,32\}$); then, the average depth of each patch is calculated, and the region with the maximum average depth is defined as the long-range region; finally, the error value is obtained by computing the absolute positional deviation between the predicted long-range region and the ground-truth long-range region. To ensure the fairness of the comparison, all comparative models adopt their officially released pre-trained weights.

**Table 5:** Error results for the long-range region on KITTI. ↓ means lower is better.

Method	Error of the long-range region in predicted depth		
	m = 8 ↓	m = 16 ↓	m = 32 ↓
DPT^10^	6.3276	1.4831	0.3281
BTS^7^	5.2024	1.2231	0.2913
Adabins^8^	4.6322	1.1050	0.3122
Simipu^38^ DepthFormer^50^	6.1814 6.1790	1.4477 1.4463	0.3431 0.3245
Ours	**4.1493**	**1.0148**	**0.2725**

**Table 6:** Boundary accuracy results on KITTI, ↑ means higher is better.

${{{t}}_{{e}}}$	Method	Precision↑	Recall↑	F1-score↑
> 0.25	DPT^10^	0.2743	0.1439	0.1866
	BTS^7^	0.2555	0.1643	0.1982
	Adabins^8^	0.2706	0.1508	0.1926
	Ours	**0.2790**	**0.1747**	**0.2130**
> 0.5	DPT^10^	0.2743	0.1439	0.1866
	BTS^7^	0.2555	0.1643	0.1982
	Adabins^8^	0.2706	0.1508	0.1926
	Ours	**0.2790**	**0.1747**	**0.2130**
> 1.0	DPT^10^	0.2834	0.0461	0.0787
	BTS^7^	0.2405	0.0536	0.0870
	Adabins^8^	0.2538	0.0516	0.0850
	Ours	**0.2891**	**0.0546**	**0.0912**

Boundary accuracy metric: Edge regions are vital for structural depth accuracy. On the KITTI dataset, we evaluate our model’s boundary performance by computing weighted Precision, Recall, and F1-score across all test images. Table 6 shows our method consistently outperforms others (bolded), with stable results at thresholds 0.25 and 0.5. This indicates our boundary predictions are accurate and robust to threshold changes. Our superior F1-scores reflect sharper, more coherent depth boundaries compared to competing methods.

### Ablation study

In the ablation study, we evaluate each core component of our framework on the KITTI datasets to validate its effectiveness.

**Table 7:** Ablation tests on the KITTI dataset, C_*i*_ represents the *i*-th block of the ResNet-50 network.

Method	Backbone	Various	δ1 ↑	δ2 ↑	δ3 ↑	REL ↓	RMSE ↓
MonoDepth2^37^	ResNet-50	–	0.952	0.992	0.998	0.0630	2.634
DPT^10^	ViT-B + R50-C1C2	–	0.959	0.995	**0.999**	0.0620	2.573
TransDepth^21^	ViT-B + ResNet-50	–	0.956	0.994	**0.999**	0.0640	2.755
Ours	Swin-L + R50-C1	– CSF CSF + MSA	0.972 0.974 **0.975**	0.997 0.997 **0.997**	**0.999** **0.999** **0.999**	0.0545 0.0515 **0.0508**	2.164 2.109 **2.107**

**Fig. 9:**
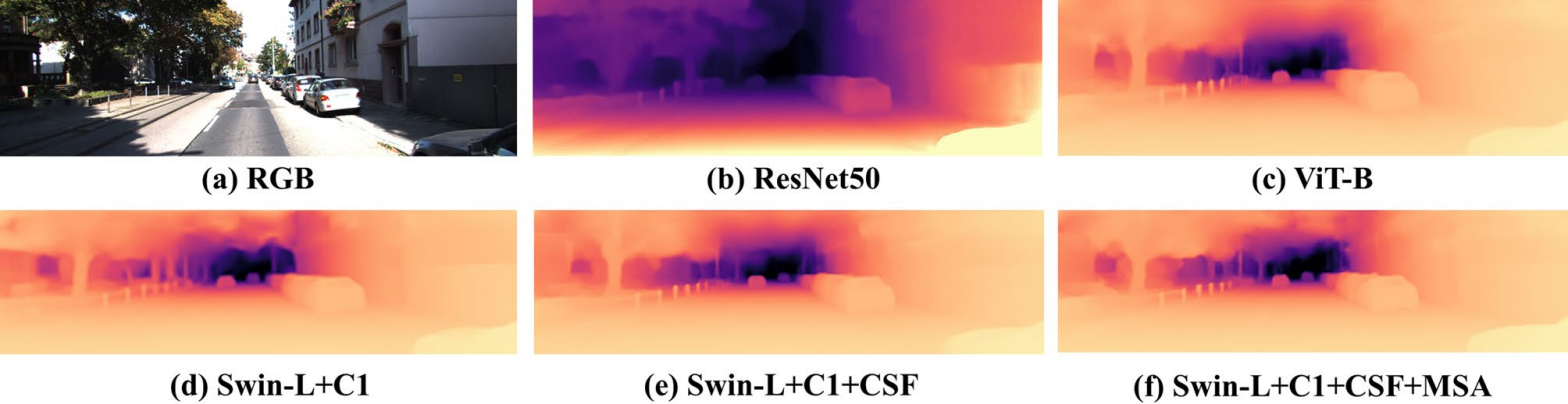
Qualitative comparison of different backbone architectures and a detailed ablation study on the contributions of the proposed CSF and MSA modules, using images from the KITTI dataset.

 Effectiveness of the heterogeneous hybrid encoder: To validate the effectiveness of the proposed heterogeneous encoder in modeling local details and long-range contextual dependencies, we compared it with typical models on the KITTI dataset. As shown in Table 7, our method significantly outperforms the pure CNN model^37^ and the pure Transformer model^10^, further confirming the general trend presented in Table 2—that hybrid architectures are more advantageous. Even when compared with TransDepth^21^ (REL = 0.058), which also adopts a hybrid design, our approach still demonstrates superior performance potential due to the effective fusion of low-level CNN features. Test results using Fig. 9a as the input image show that the qualitative results in Fig. 9b–d further corroborate the aforementioned conclusion: our model not only achieves higher accuracy in the depth estimation of distant vehicles but also accurately preserves the fine contours of objects.

**Fig. 10:**
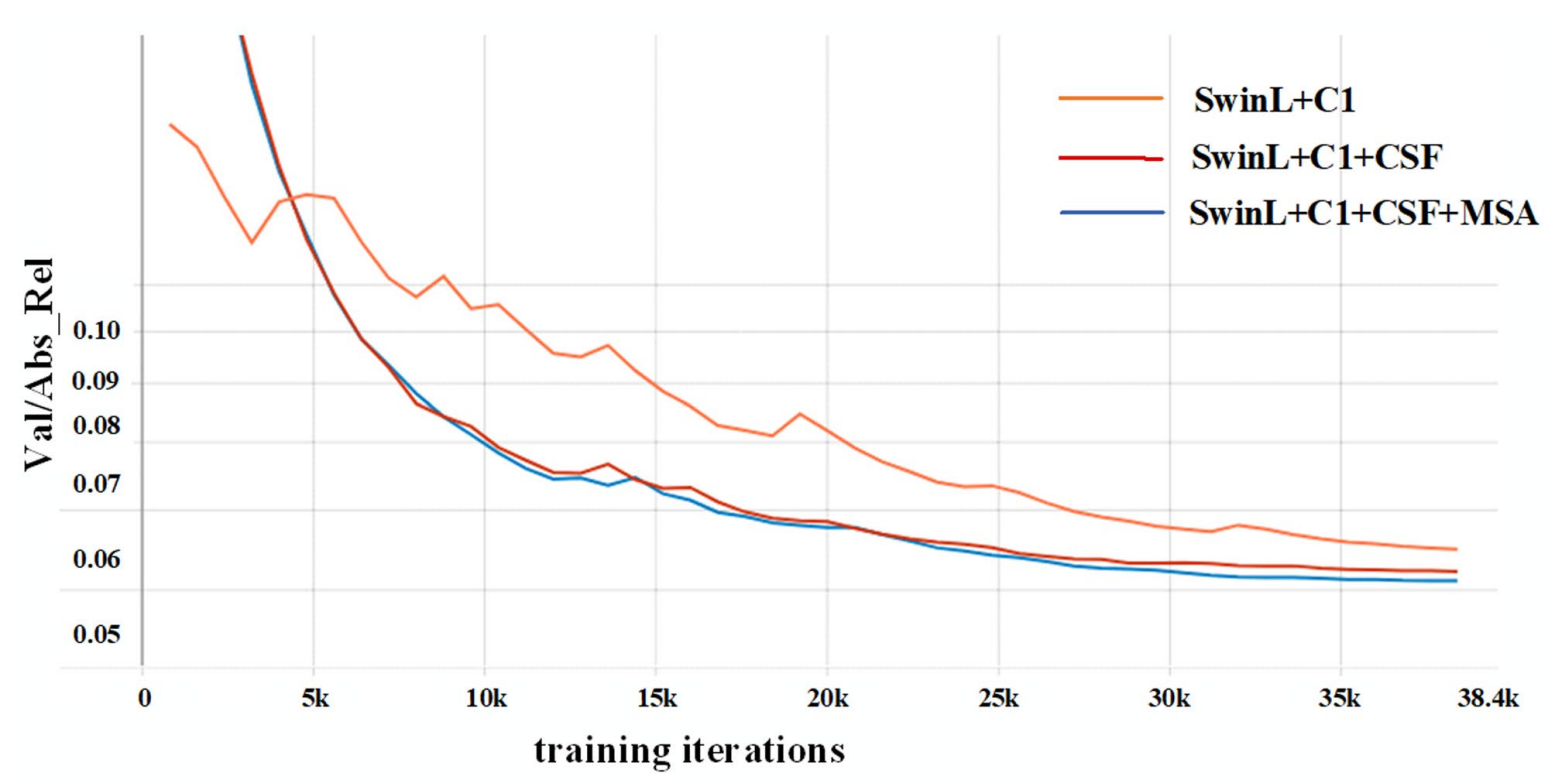
Ablation study of key components on the KITTI dataset.

Evaluation of the CSF Module: The introduction of the CSF module into the heterogeneous encoder framework effectively enhances feature aggregation capability. As shown in Table 8, each sub-module of CSF independently improves performance, with their combination achieving the best results: compared to the baseline model, CSF improves Abs Rel by 0.003 and reduces RMSE by 0.021. Table 9 further demonstrates that the module significantly reduces depth errors in long-range regions through multi-scale feature fusion. Comparing Fig. 9d (without CSF) and Fig. 9e (with CSF), it can be observed that CSF markedly enhances the model’s ability to represent distant targets, with textures of objects such as road studs appearing clearer. Figure 10 further confirms that the inclusion of CSF notably improves estimation accuracy. These results indicate that the module overcomes the limitations of traditional single-dimensional fusion methods.

**Table 8:** Ablation analysis of CSF submodules on KITTI dataset. √ denotes the use of different submodules within the CSF module.

Backbone	Various			δ1 ↑	REL ↓	RMSE ↓
	GLC	MSPM	CCF			
Swin-L + R50-C1				0.972	0.0545	2.163
	√			0.970	0.0551	2.156
		√		0.972	0.0543	2.142
			√	0.972	0.0540	2.149
		√	√	0.972	0.0533	2.156
	√	√	√	**0.973**	**0.0519**	**2.142**

**Table 9:** CSF for long-range region error assessment on KITTI dataset.

Method			Error of the long-range region in predicted depth		
GLC	MSPM	CCF	m = 8 ↓	m = 16 ↓	m = 32 ↓
–	–	–	5.7934	1.3323	0.3083
√	√	√	**4.1493**	**1.0148**	**0.2725**

**Fig. 11:**
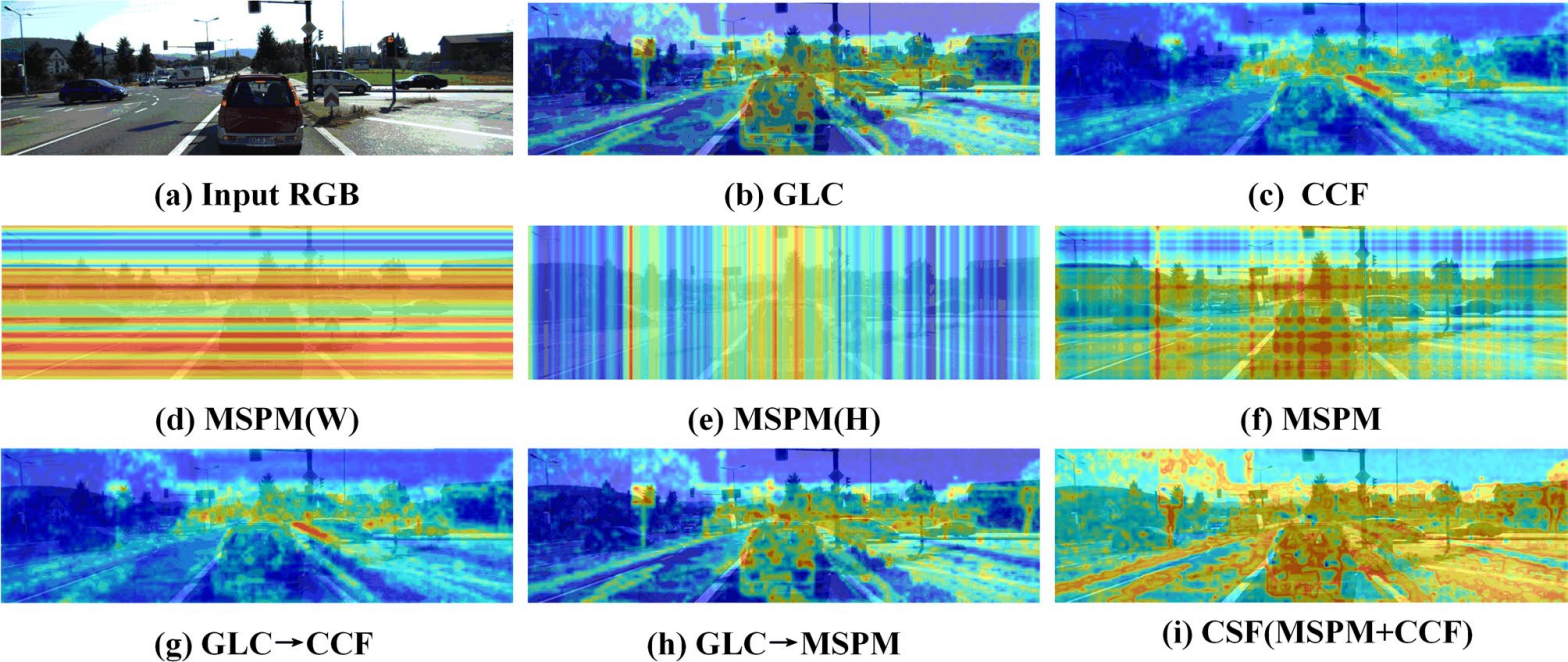
Visualizes the attention heatmap of the CSF module on KITTI.

To qualitatively validate the effectiveness of the CSF module, we visualize the attention weights of its three submodules in the form of heatmaps using test images (Fig. 11a) from the KITTI dataset (results from one feature layer are presented here). As shown in Fig. 11, the heatmap of the GLC submodule (Fig. 11b) demonstrates that the network can simultaneously focus on nearby vehicles and medium-to-long-range targets, effectively balancing local details and global information. The MSPM submodule exhibits directional awareness: its horizontal attention (Fig. 11d) focuses on horizontal structures such as roads, while its vertical attention (Fig. 11e) captures vertical contours like vegetation and buildings. Their fusion result (Fig. 11f) clearly distinguishes different regions. The heatmap of the CCF submodule (Fig. 11c) shows uniform coverage across near, medium, and distant scenes, reflecting its ability to effectively integrate cross-scale features. According to the overall mechanism of the CSF module (Fig. 1), the process can be summarized as follows: GLC preprocessing (Fig. 11b) first enhances the response in key regions; the information then flows in parallel through the MSPM branch (Fig. 11h) and the CCF branch (Fig. 11g), which respectively reinforce spatial structure and supplement multi-scale details; finally, complementary feature enhancement is achieved through gated fusion (Fig. 11i).

**Table 10:** Quantitative comparison with other modules on KITTI.

Backbone	Blocks	δ1 ↑	Sq_rel↓	Log_10↓	REL ↓	RMSE ↓
Swin-L + R50-C1	MSCA^51^	0.973	0.161	0.0238	0.0552	2.134
	Swin LKA^13^	0.972	0.160	0.0238	0.0538	2.149
	EMCAM^17^	0.972	0.159	0.0231	0.0534	2.139
	CCFF^18^	0.971	0.158	0.0235	0.0540	2.163
	SMFA^52^	0.972	0.163	0.0236	0.0546	2.152
	CBAM^53^	0.972	0.164	0.0235	0.0547	2.159
	SCSA^54^	0.972	0.161	0.0235	0.0543	2.142
	Ours (CSF)	**0.974**	**0.152**	**0.0225**	**0.0515**	**2.107**

We also compared our CSF module with other attention modules. To ensure a fair comparison, only the CSF module was replaced with other attention modules for evaluation. We compared against various channel attention, spatial attention, and multi-scale feature fusion attention modules proposed in recent years. As shown in Table 10, our method outperforms all other modules.

**Table 11:** Ablation study of key decoder components on KITTI dataset: comparison of different upsampling modules and the effect of including the MSA module.

Decoder	δ1 ↑	REL ↓	RMSE ↓
Nearest-neighbor upsampling	0.973	0.0519	2.142
Nearest-neighbor upsampling + MSA	**0.975**	0.0510	2.110
DSUB	0.974	0.0515	2.109
DSUB + MSA	**0.975**	**0.0508**	**2.107**

By integrating the MSA module with DSUB, we further enhanced detail recovery and spatial consistency during the decoding stage. As shown in Table 11, this combined approach reduces the REL metric to 0.0508 and improves the δ1 metric to 0.975. The qualitative results in Fig. 9e,f show that the MSA module notably improves the prediction accuracy for distant vehicles. Meanwhile, the results in Fig. 10 demonstrate that the attention mechanism plays a key role in strengthening feature representation by accelerating model convergence.

**Fig. 12:**
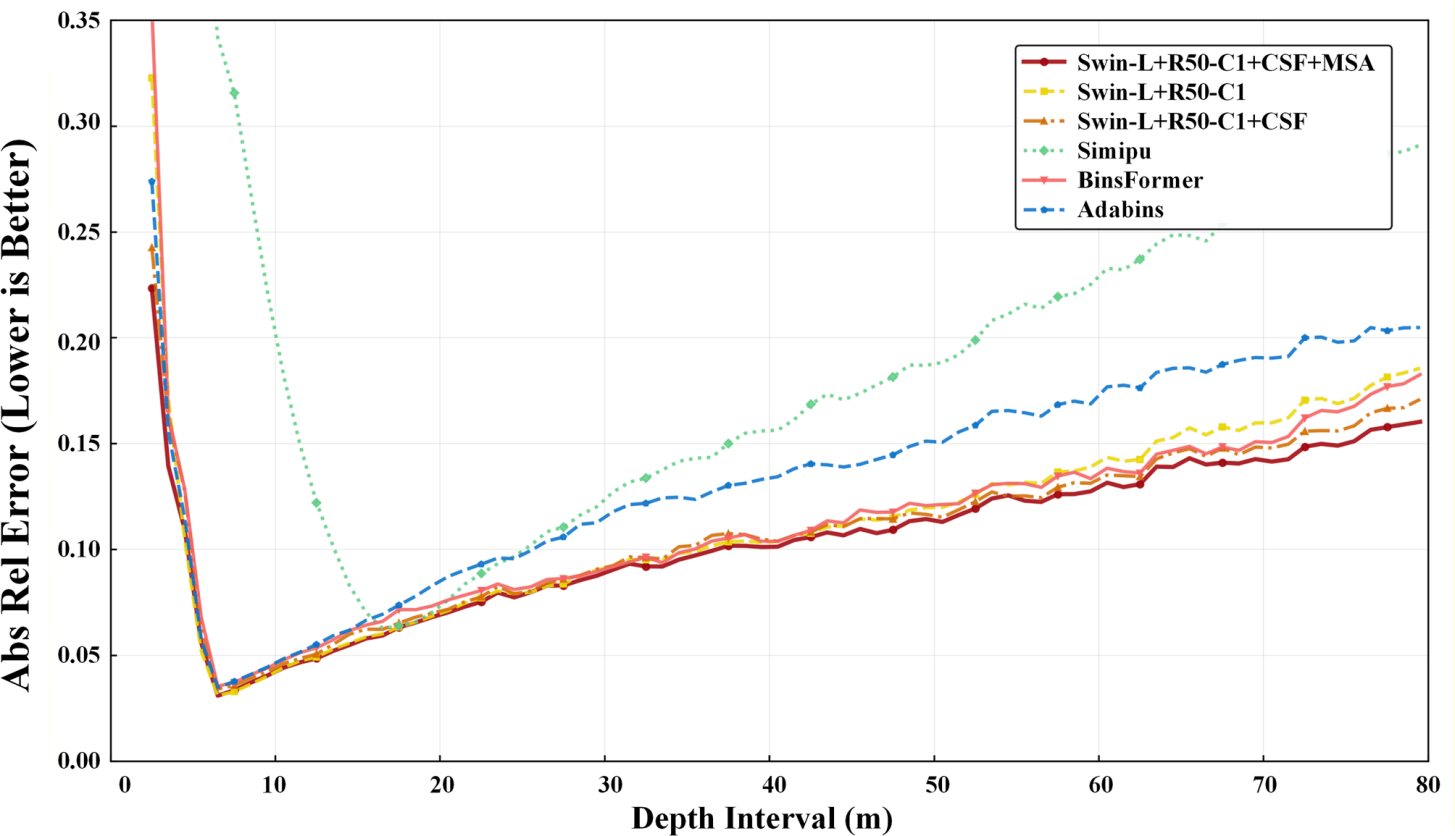
Fine-grained quantitative results of our pilot study on the KITTI dataset. We divide the depth range (0–80 m) into 80 intervals. Point (*i; j*) in the plot represents the Abs-Rel of the model is j on depth interval (*i; i* + 1] m.

Long-range performance details: To evaluate the model’s performance in long-range scenarios, we divided the test depth range into three intervals: near range (0–20 m), long range (60–80 m), and full range (0–80 m), and calculated the Abs-Rel error for each interval. As shown in Table 12, compared with classical methods such as ResNet50 and ViT, our method achieves consistent superiority across all intervals, with the most notable improvement in the long-range (60–80 m) interval—validating the effectiveness of our model for long-range depth estimation. The discrepancy between the 0–80 m evaluation results and those in Table 2 stems from the statistical granularity: Table 2 employs pixel-level global statistics, while Table 12 uses sample-level mean statistics. Furthermore, we evenly partitioned the depth range into 80 intervals and plotted the variation curve of Abs-Rel error against distance in Fig. 12. The results indicate that our method performs well over the entire distance range.

**Table 12:** More detailed ablation quantitative results on the KITTI dataset.

Method	Backbone	Range(m)	δ1 ↑	δ2 ↑	δ3 ↑	REL ↓	RMSE ↓
He et al.^1^	ResNet50	0–20	0.973	0.998	1	0.054	0.985
		60–80	0.600	0.900	0.972	0.188	14.30
		0–80	0.952	0.994	0.999	0.065	2.596
Dosovitskiy et al.^55^	ViT	0–20	0.955	0.995	0.999	0.071	1.275
		60–80	0.727	0.936	0.985	0.150	11.86
		0–80	0.938	0.992	0.999	0.080	2.695
AdaBins^8^	EFN-B5 + ViTmini	0–20	0.974	0.996	1	0.053	1.001
		60–80	0.569	0.897	0.975	0.192	14.68
		0–80	0.943	0.991	0.998	0.068	3.085
Binsformer^11^	Swin-L	0–20	0.976	0.997	0.999	0.052	0.988
		60–80	0.719	0.939	0.984	0.152	12.03
		0–80	0.957	0.994	0.999	0.062	2.627
Simipu^38^	-	0–20	0.596	0.972	0.996	0.219	2.113
		60–80	0.357	0.785	0.944	0.258	19.03
		0–80	0.631	0.965	0.994	0.203	4.034
Ours	Swin-L + R50-C1	0–20	0.979	0.998	1	0.050	0.982
		60–80	0.739	0.942	0.984	0.148	11.83
		0–80	0.961	0.995	0.999	0.061	2.577
	Swin-L + R50-C1 + CSF	0–20	0.982	0.998	1	0.047	0.911
		60–80	0.735	0.949	0.984	0.147	11.63
		0–80	0.963	0.995	0.999	0.058	2.550
	Swin-L + R50-C1 + CSF + MSA	0–20	0.983	0.998	1	0.046	0.906
		60–80	0.720	0.945	0.983	0.152	11.95
		0–80	0.963	0.995	0.999	0.057	2.523

**Fig. 13:**
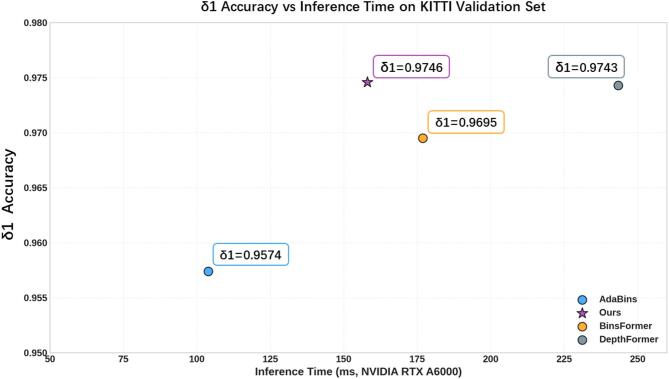
Accuracy δ1 vs. Inference Time on KITTI validation set. The speed is measured using a single GPU.

Inference time evaluation: In addition to the accuracy (δ1) of depth prediction, inference speed is also a critical metric. For this purpose, we evaluated the inference time of our model on the KITTI validation set. To ensure a fair comparison, all other baseline models were tested using their officially released public weights. As shown in Fig. 13, our method achieves a good balance between accuracy and speed.

## Conclusion

To tackle the local-global feature imbalance, inefficient fusion, and detail loss in long-range monocular depth estimation, this study proposes a high-performance framework with three core innovations. A heterogeneous hybrid encoder combines ResNet-50’s initial convolutions and Swin Transformer’s hierarchical attention to balance local texture extraction and long-range dependency modeling. The CSF module realizes precise binding of distant semantic features and local edges via multi-scale and spatial-channel coupling. The attention-driven decoder with DSUB and MSA optimizes detail restoration and spatial consistency. Experiments on KITTI and SUN RGB-D validate its superiority and strong generalization, advancing long-range depth estimation for robotics and UAV perception. Future work will enhance environmental robustness, optimize lightweight designs for real-time deployment, and explore multi-modal data fusion to break pure visual accuracy limits.

## Supplementary Information

Below is the link to the electronic supplementary material.

Supplementary Material 1

## Data Availability

All data generated or analyzed during this study are included in this published article. We provide the proposed model code at: https://github.com/sarielbit/The-Long-Range-Depth-Estimation.
